# Effectiveness of Physical Exercise on Indicators of Metabolic Syndrome in Adults: A Systematic Review with Meta-Analysis of Clinical Trials

**DOI:** 10.3390/jfmk10030244

**Published:** 2025-06-27

**Authors:** Brandon Galván, Liliana Aracely Enriquez del Castillo, Luis Alberto Flores, Estefania Quintana-Mendias, Flor Isela Torres-Rojo, Cinthia Verónica Villegas-Balderrama, Natanael Cervantes-Hernández

**Affiliations:** 1 Faculty of Physical Culture Science, Autonomous University of Chihuahua, Chihuahua 31109, Mexico; a314361@uach.mx (B.G.); lolivares@uach.mx (L.A.F.); esquintana@uach.mx (E.Q.-M.); vvillegas@uach.mx (C.V.V.-B.); 2 Faculty of Chemical Science, Autonomous University of Chihuahua, Chihuahua 31109, Mexico; fitorres@uach.mx

**Keywords:** aerobic exercise, endurance exercise, resistance training, exercise training, metabolic diseases, glucose metabolism disorders, insulin resistance, hyperinsulinism

## Abstract

**Background:** The increased presence of metabolic syndrome (MetS) indicators globally is considered a public health problem, and the dose-response of exercise is not clarified. **Objectives:** This purpose of this study was to analyze the effectiveness of changes in biochemical, physiological, and anthropometric indicators of MetS based on distinct types of exercise in adults. **Methods**: Based on PRISMA guidelines, we searched the following databases—PubMed, Cochrane, LILACS, Springer Link, and Science Direct— for clinical trials investigating the effect of exercise in MetS indicators, without date or language restrictions. The quality of evidence and risk of biases were assessed using the PEDro scale. The impact of aerobic training “AT,” resistance training “An-T”, concurrent training “CT”) on MetS indicators (body mass index (BMI), waist circumference (WC), total body weight (TBW), total cholesterol (TC), LDL-c, HDL-c, fasting blood glucose (FBG), triglycerides (TG), and systolic and diastolic blood pressure (SBP, DBP)) were included in this analysis. **Results**: A total of 10 clinical trials was selected. Results demonstrate a heterogeneity of over 50% (*p* < 0.001). A mean difference was found for TC by AT (−23.70 mg/dL, *p*= 0.003) and An-T (3.91 mg/dL, *p*= 0.003); on HDL by CT (0.12 mg/dL, *p* = 0.004); on FBG by AT (−0.66 mg/dL, *p* = 0.02), CT (−1.42 mg/dL, *p* = 0.01); on DBP by AT (−0.79 mmHg, *p* = 0.02). **Conclusions**: There is a dominance of concurrent exercise over other types of exercise, considering the greater effectiveness and significance of the effect of seven MetS indicators, including HDL and fasting blood glucose, with a major effect size.

## 1. Introduction

Globally metabolic syndrome (MetS) is a clinical disorder characterized by the presence of comorbidities or risk factors that may or may not interact with each other, is currently considered a public health problem [1], and includes diseases such as abdominal obesity, type 2 diabetes mellitus (T2DM), hypertension and dyslipidemia, as well as mechanisms related to the pathogenesis of MetS [2]. Therefore, it can be developed by hereditary genetic factors and environmental factors. Among environmental factors, lifestyle is a key determinant for the appearance of MetS, so physical inactivity or lack of exercise, high sedentary behavior, and inadequate nutrition promote obesity, representing the first instance of the incidence of MetS. The presence of obesity causes functional alterations in adipose tissue induced by an excess percentage of body fat, which contributes to the secretion of inflammatory hormones such as IL6, TNF and leptin, which act as promising biomarkers for the prognosis of non-communicable diseases associated with metabolic syndrome [3,4,5].

Its incidence is considered primary by combining a set of biochemical, physiological, and anthropometric abnormalities that co-occur, followed by the incidence of obesity and T2DM [6], so quantifying comparative data related to MetS is complicated since its epidemiology depends on a coarse criterion; this is usually interpreted twice as high as T2DM, estimating MetS’s prevalence as approximately a quarter of the world’s adult population [7,8]. The prevalence of MetS components in the world ranges between 22% and 47% due to age, sex, and ethnicity [9]. In Mexico, according to the aggrupation of prevalences through the criteria of organizations such as the Adult Treatment Panel III (ATP III), the International Diabetes Federation (IDF), the World Health Organization (WHO), and the American Heart Association (AHA)/National Heart, Lung, and Blood Institute (NHLBI), MS is 41% with a variation of 23% of its adult population; this depends on the present indicators [6].

Physical exercise induces physiological adaptations depending on the type and intensity of exercise performed. Concerning aerobic exercise, it has been shown to promote weight loss and an increase in lipolysis through the activation of AMPK (AMP-activated protein kinase) and the stimulation of fatty acid oxidation, which favors the reduction of visceral adipose tissue, which sequentially causes the release of adiponectin, an adipokine with anti-inflammatory and insulin-sensitizing effects, which positively modulates lipoprotein levels, raising HDL and reducing LDL concentrations, as well as stimulating uptake in peripheral tissues. These adaptations contribute to the control of dyslipidemia, insulin resistance, and chronic low-grade inflammation; on the other hand, anaerobic exercise, particularly strength training, stimulates protein synthesis produced by activating the mTOR (mechanistic target of rapamycin) pathway, induced by the contraction of a large number of motor units. This process promotes muscle hypertrophy, increases resting energy expenditure, and reduces body fat percentage. It also improves glucose uptake in skeletal muscle independently of insulin, which is essential for glycemic control in people with type 2 diabetes and for preventing the progression of metabolic syndrome. Together, both types of exercise act through complementary mechanisms that are strategic in the prevention and treatment of non-communicable diseases [10].

Likewise, a concurrent type of exercise is characterized by combining both in a single routine, which can predict greater effectiveness and time efficiency in developing muscular strength and aerobic capacity compared to resistance training alone. The scientific literature has established that concurrent exercise may generate positive modulatory effects on markers of non-communicable diseases because it offers the benefits of aerobic-type exercise for cardiovascular markers as well as those of strength work for metabolic diseases; however, when investigating in depth the results obtained by exercise programs of a single type of training, it is common to observe that there is no greater effect related to the decrease in these indicators due to the diversity in the programming of training, such as total volume and not only training. Added to this, a variability of biochemical abnormalities that diverge simultaneously is complicated to control, so establishing the effectiveness of this versatility in exercise programs, considering populations with similar conditions, is essential for a more efficient exercise prescription that allows regulating diseases in a safe way. The findings of the interference phenomenon show that combined training results in uncompromised gains in muscular power, with no compromise in the development of aerobic capacity; otherwise, the manipulation of training intensity and volume are inversely related, where normally as intensity increases, volume decreases [11].

There is well-established evidence that aerobic exercise significantly enhances aerobic capacity, which is positively associated with cardiopulmonary and metabolic parameters while also contributing to the reduction in cardiovascular disease (CVD) risk factors. In contrast, resistance training more effectively improves endurance, muscular strength, and muscle mass. Furthermore, substantial evidence suggests that resistance-trained muscle may lead to greater improvements in glucose regulation compared to aerobic training, particularly regarding body composition, glycemic control, and insulin sensitivity and signaling. In this context, concurrent exercise (combining resistance and aerobic training) may further optimize metabolic outcomes and reduce CVD risk factors [12,13,14].

The chronic implications present in MetS increase treatment costs, this being the first level of health care. The demand for medical care significantly influences health sector costs, directly affecting the amount for medications up to a possible surgical intervention; expenses of MXN 50,000 per person annually in the Mexican population are estimated [15,16]. By 2040 in Mexico, the peak mortality is estimated to be between 77.5 and 80 years; this will be influenced by the control of non-communicable diseases, which complicates the future outlook because despite having 5.1% of the GDP (gross domestic product) allocated to health [17], the balance is insufficient to subsidize hospital costs [18].

Based on the above, a systematic review with meta-analysis was conducted to analyze the effectiveness of changes in biochemical, physiological, and anthropometric indicators of MetS based on distinct types of exercise in adults.

## 2. Materials and Methods

### 2.1. Search Strategy

This systematic review was conducted according to PRISMA guidelines and following the PICO strategy. The question for the PICO strategy was “What is the optimal dose-response of different types of physical exercise to achieve clinically significant improvements in the main indicators of metabolic syndrome in adults?” We use P = adults; I = exercise programs; C = type of the exercise programs; O = clinical data of each indicator post exercise. Digital databases used were PubMed, Springer Link, Science Direct, Cochrane, and LILACS. The search was carried out during June 2025 without date or language restrictions. The search strategy was established using the Boolean operators AND and OR. The connected keywords were “Physical activity program type” AND “Exercise program type” AND “Metabolic syndrome” NOT “Diet” in their different combinations. For the PubMed database, the filters used were clinical trial, controlled trial, humans, adult: 19+ years, adult: 19–44; for Springer Link, the filters used were article, open access, metabolic syndrome; for Science Direct, the filters used were research articles, medicine and dentistry, nursing and health professions, biochemistry, genetics and molecular biology, diabetes and metabolic syndrome: clinical research and reviews; for Cochrane, filters were not used.

### 2.2. Study Selection and Selection Criteria

The selection process was initially carried out by title (n = 329); if it was a duplicate tittle, an Excel was used to eliminate it, and (2) if the title was relevant, the article summary was read (n = 14). Once the search objective was met, the article was read to determine its inclusion in the systematic review and meta-analysis, considering the inclusion and exclusion criteria. A total of 329 articles met the following inclusion criteria: (a) exercise program implementation, (b) applied to adults aged 18 to 64, (c) clinical trial-type experimental design, and (d) evaluated at least three MetS indicators. Exclusion criteria were (a) intervention with a sports focus and (b) changes in your usual diet. The selected articles were added with an ID, starting with apostrophe “A” followed by a number according to their order of appearance.

### 2.3. Data Extraction

The following data were extracted from the included trials: (a) citation and country, (b) objective, (c) population characteristics, (d) exercise program characteristics, (e) post-intervention outcomes about body mass index, waist circumference, body weight, total cholesterol, LDL, (f) HDL, fasting blood glucose, triglycerides, systolic blood pressure, and diastolic blood pressure.

### 2.4. Methodological Quality

The methodological quality of the included studies was evaluated using the Physiotherapy Evidence Database (PEDro) scale, which assigns scores based on the number of items met in a clinical trial. It is important to note that the scale excludes the blinding of subjects and applicators due to the nature of the study (items 3, 5, 6, 7), resulting in a maximum score of seven points for the remaining. The PEDro scale assigns higher scores to studies with higher methodological quality. The classification used is as follows: scores ranging from 0 to 3 were rated “poor”, scores between 4 and 5 were rated as “fair”, and scores ranging from 6 to 7 were categorized as “high” [19].

### 2.5. Statistical Analysis

The effect size was estimated for each MetS indicator by evaluating at least four studies based on calculating the weighted mean difference (WMD) with a 95% confidence interval (CI). The WMD and effect size were visually presented in the forest plot. Heterogeneity was estimated, with an I^2^ value ≥ 50%, and low or homogeneous, with an I^2^ value < 50%, using the random-effects or fixed-effects models, respectively. All analyses were performed in Review Manager version 5.4, considering a significance of *p* ≤ 0.05. [20].

## 3. Results

### 3.1. Search Results

Four databases were selected from the 329 articles found through the keyword search. Twenty articles were found in PubMed, one hundred ninety-seven in Springer, forty-seven in Science Direct, fifty-five in Cochrane, and four in LILACS. Of these, three hundred seventeen articles were discarded because they did not meet the inclusion criteria, two were excluded due to duplication, and only 10 articles were selected for the meta-analysis. These articles included human participants and physical exercise interventions. The article selection process can be seen in the flowchart [21,22,23,24,25,26,27,28,29,30].

### 3.2. Characteristics of the Selected Studies

#### 3.2.1. Results of Methodological Quality

The methodological quality through the PEDro scale (Table 1) allowed zero articles to be classified as bad; two articles were considered fair [22,26], and eight articles were considered good [21,23,24,25,27,28,29,30]. The overall average was good, with an average of six [19].

#### 3.2.2. Characteristics of the Included Studies

Table 2 shows the characteristics of the studies. A total of 12 controlled clinical trial studies, of which 10 are randomized, were included in the meta-analysis. The total number of n was 1454 adults of both sexes, with a mean age of 61.40 years, and shared pathologies associated with MS, such as cardiovascular disease (CVD) and coronary artery disease (CAD).

#### 3.2.3. Characteristics of Exercise Programs

A total of 21 physical exercise programs were found in the 12 selected studies, which were different from each other, so using the FITT principle they were classified as follows: 1. Frequency: two times per week (A7, A8, A12, A14, A15, A16), three times per week (A1, A3, A4, A9, A10, A11, A13), five times per week (A5, A6, A17), daily (A2). 2. Intensity: unspecified (A1, A2, A6, A12, A13, A16), 85–95% HRpeak (A3, A4), 60–70% HRpeak (A5, A8, A14), 55% HRpeak (A7), 50–80% HRpeak (A9, A10), 70–90% HRpeak (A11), 50% 1RM (A15), 40–60% HRR (A17). 3. Type: aerobic (A1, A3, A4, A5, A6, A7, A8, A9, A10, A11, A12, A13, A14, A15, A17), anaerobic (A7, A8, A9, A10, A13), unspecified (A2, A16). 4. Time session: 16–30 min (A3, A5), 31–45 min (A1, A4, A11, A17), 46–60 min (A2, A6, A9, A10, A13), 61–75 min (A7, A8, A14, A15), 180 min (A12, A16).

### 3.3. Effects of Exercise on Indicators of Metabolic Syndrome

**Figure 1:**
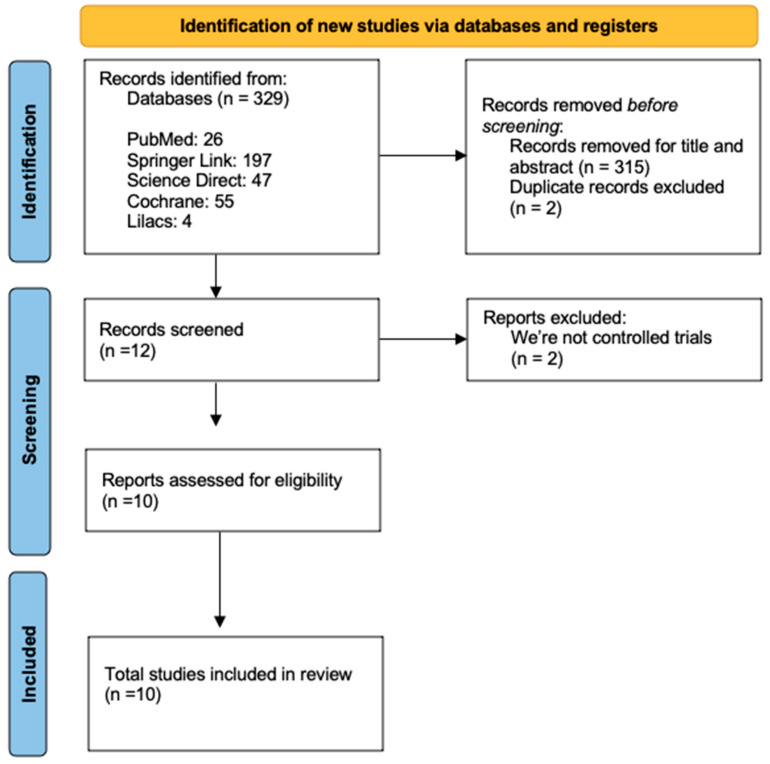
Methodology flow chart.

Table 2 summarizes the effects of exercise on METs indicators found in the included studies. A meta-analysis was conducted to evaluate the impact of interventions and exercise on indicators of metabolic syndrome, as shown in Figure 1.

**Total cholesterol**

**Figure 2:**
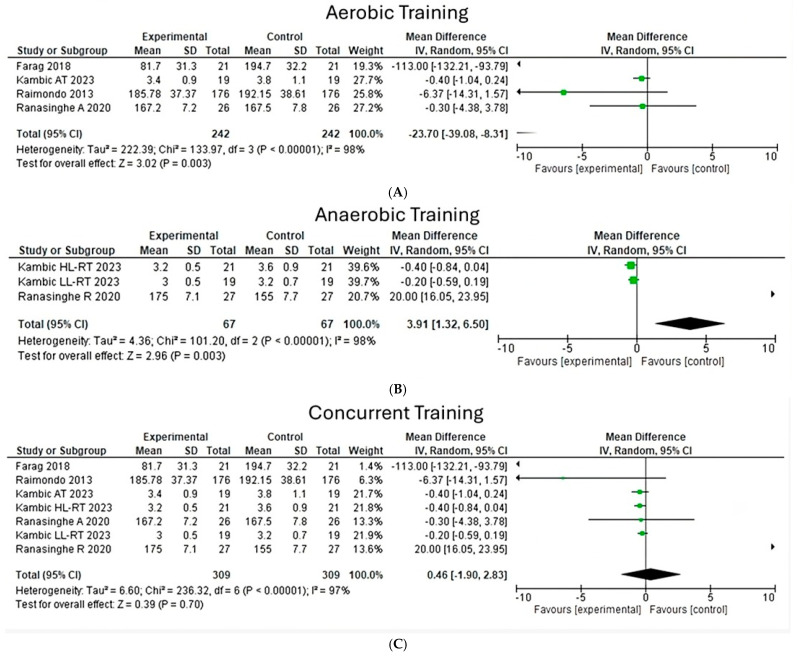
Total Cholesterol under the effect of different exercises program. (**A**) Total cholesterol under effect of aerobic training. (**B**) Total cholesterol under effect of anaerobic exercise. (**C**) Total cholesterol under effect of concurrent training [24,26,27,28].

When analyzing the effects of different types of exercise on total cholesterol, it is possible to observe that a heterogeneity of 98% is shown (Chi^2^ = 133.97, *p* < 0.00001) (Figure 2A) in four studies of aerobic exercise; on the other hand, in three studies with anaerobic exercise, a heterogeneity index of 98% was observed (Chi^2^ = 101.20, *p* < 0.00001) (Figure 2B). It is worth noting that in both types of exercises, the overall effect size is significant, with a z value of 3.02 (*p* = 0.003) for aerobic exercise. Likewise, a z value of 2.96 (*p* = 0.003) was found for anaerobic exercise, while the effect determined by concurrent exercise was not significant (z = 0.39, *p* = 0.70) (Figure 2C); it should be noted that the studies with the most important weight are those of Kambic and Ranasinghe.

**Low- and high-density lipoprotein**

**Figure 3:**
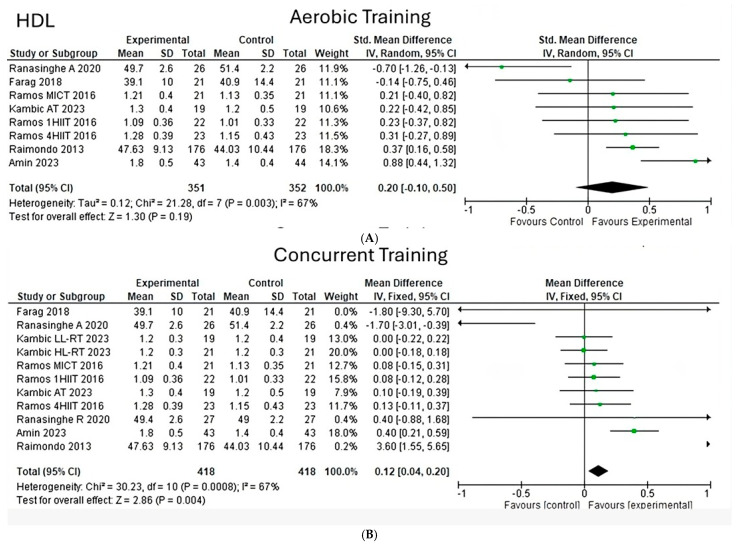
High density lipoprotein under the effect of different exercises program. (**A**) High density lipoprotein under the effect of aerobic training. (**B**) High density lipoprotein under the effect of concurrent training [24,25,26,27,28,30].

Upon analyzing high-density lipoproteins (HDL) under aerobic training, it can be observed that a heterogeneity of 67% is shown (Chi^2^ = 21.28, *p* < 0.003) (Figure 3A) in eight studies. Likewise, in 11 interventions of two types of training, a heterogeneity of 67% is shown (Chi^2^ = 30.23, *p* < 0.00008) (Figure 3B), highlighting that the size of the general effect is significant with a value (z = 2.86, *p* = 0.004) for a concurrent exercise, while for an aerobic exercise a value (z = 1.30, *p* = 0.19) is shown; the most significant weight is found in the studies of Kambic and Raimondo.

**Figure 4:**
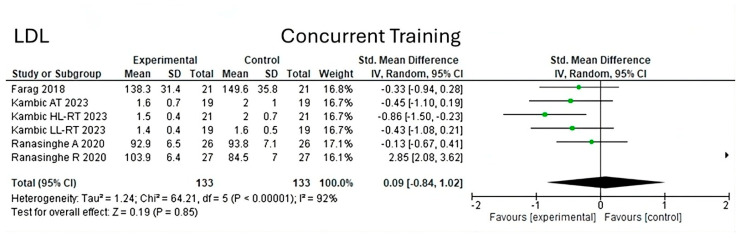
Low density lipoprotein under the effect of concurrent training [24,27,28].

On the other hand, on analyzing low-density lipoproteins (LDL) under concurrent training conditions, a heterogeneity of 92% (Chi^2^ = 64.21, *p* < 0.00001) (Figure 4) can be observed in six studies, determining the overall effect size with a value (z = 0.19, *p* = 0.85).

**Fasting blood glucose**

**Figure 5:**
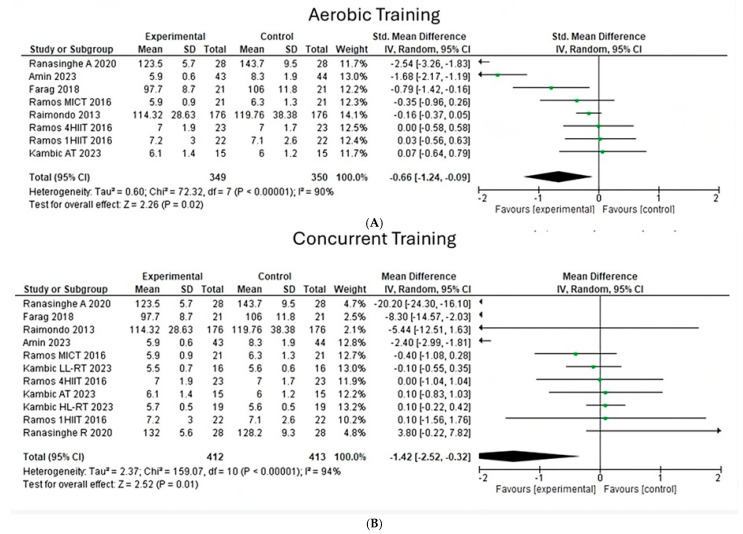
Fasting blood glucose under the effect of different exercises program. (**A**) Fasting blood glucose under the effect of aerobic training. (**B**) Fasting blood glucose under the effect of concurrent training [24,25,26,27,28,30].

When analyzing the effects of aerobic and concurrent exercises on fasting glycemia, a heterogeneity of 90% (Chi^2^ = 72.32, *p* < 0.00001) (Figure 5A) is observed in eight aerobic exercise studies, while in 11 concurrent studies, a heterogeneity index of 94% was observed (Chi^2^ = 159.07, *p* < 0.00001). Thus, it is highlighted that in both types of exercise, the overall effect size is significant, with a value (z = 2.26, *p* = 0.02) for aerobic exercise and a value (z = 2.52, *p* = 0.01) for concurrent exercise. Likewise, in aerobic-type interventions, the Raimondo study has a greater weight, while in the concurrent type, the most significant weight is found in Kambic’s studies.

**Diastolic blood pressure**

**Figure 6:**
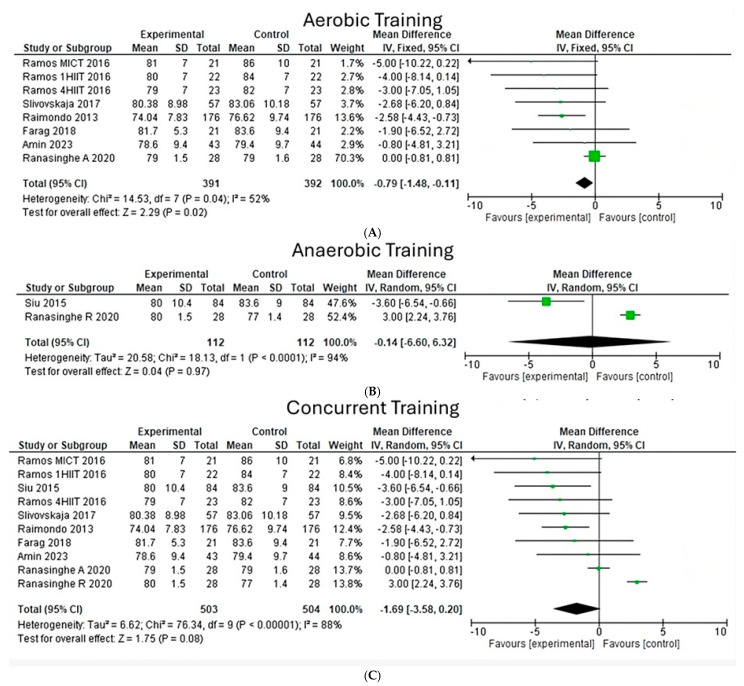
Diastolic blood pressure under the effect of different exercises program. (**A**) Diastolic blood pressure under the effect of aerobic training. (**B**) Diastolic blood pressure under the effect of anaerobic training. (**C**) Diastolic blood pressure under the effect of concurrent training [22,23,24,25,26,28,30].

Upon analyzing the effects of different types of exercise on diastolic blood pressure, a heterogeneity of 52% was observed (Chi^2^ = 15.53, *p* < 0.04) (Figure 6A) in eight aerobic studies, where the greatest weight is found in the study of Ranasinghe. On the other hand, in two anaerobic studies, a heterogeneity of 94% was observed (Chi^2^ = 18.13, *p* < 0.00001), highlighting that the overall effect size in aerobic exercise is significant with a value (z = 2.29, *p* = 0.02), while for anaerobic exercise, it was not significant with a value (z = 0.04, *p* = 0.97). Likewise, a value was determined for concurrent exercise (z = 1.75, *p* = 0.08), marking a trend, where the greatest weight is found in studies by Ranasinghe.

**Body mass index**

**Figure 7:**
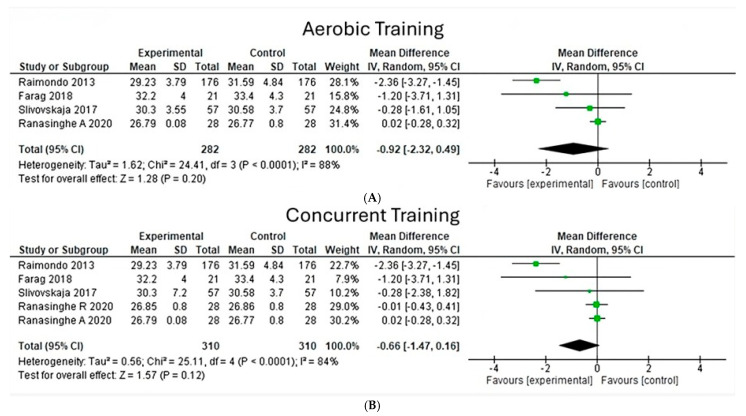
Body mass index under the effect of different exercises program. (**A**) Body mass index under the effect of aerobic training. (**B**) Body mass index under the effect of concurrent training [22,24,26,28].

Upon analyzing the effects of aerobic and concurrent exercises on body mass index, it is possible to observe a heterogeneity of 88% (Chi^2^ = 24.41, *p* < 0.00001) (Figure 7A) in four aerobic-type studies; on the other hand, five concurrent exercise studies show a heterogeneity of 84% (Chi^2^ = 25.11, *p* < 0.00001) (Figure 7B), with Ranasinghe’s studies standing out in both for their greater weight. The overall effect size in both exercise types was insignificant, having a value (z = 1.28, *p* = 0.20) for aerobic exercise and a value (z = 1.57, *p* = 0.12) for concurrent exercise.

**Total body weight**

**Figure 8:**
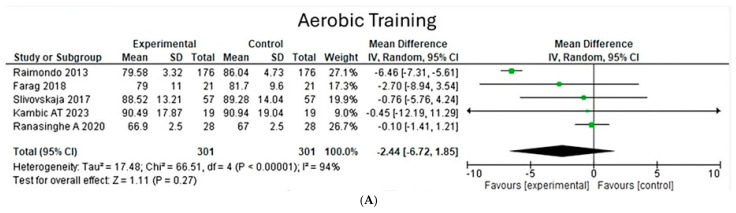

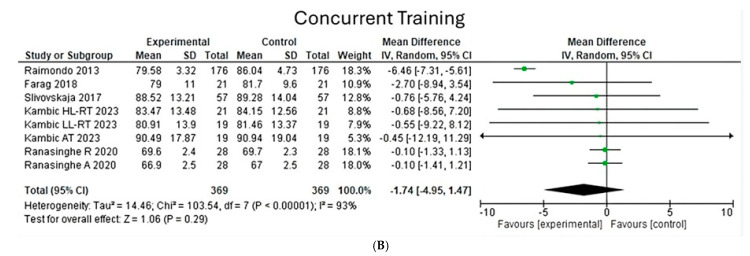
Total body weight under the effect of different exercises program. (**A**) Total body weight under the effect of aerobic training. (**B**) Total body weight under the effect of concurrent training. [22,24,26,27,28].

Analyzing the effects of aerobic and concurrent training conditions on total body weight shows a heterogeneity of 94% (Chi^2^ = 66.51, *p* < 0.00001) in five aerobic studies, with a greater weight found in studies by Ranasinghe and Raimondo; on the other hand, in eight concurrent studies, a heterogeneity of 93% was observed (Chi^2^ = 103.54, *p* < 0.00001), with Ranasinghe’s studies standing out for their greater weight. Both types of exercise do not present a significance in terms of the size of the general effect, with a value (z = 1.11, *p* = 0.27) for aerobic exercise and a value (z = 1.06, *p* = 0.29) for concurrent exercise (Figure 8).

**Indicators unchanged**

Those indicators not mentioned in the previous results, such as triglycerides, systolic blood pressure, and waist circumference, did not show favorable significance. Likewise, since they did not provide sufficient data to perform an analysis, they were discarded from the results, and therefore from the meta-analysis.

**Systolic blood pressure**

**Figure 9:**
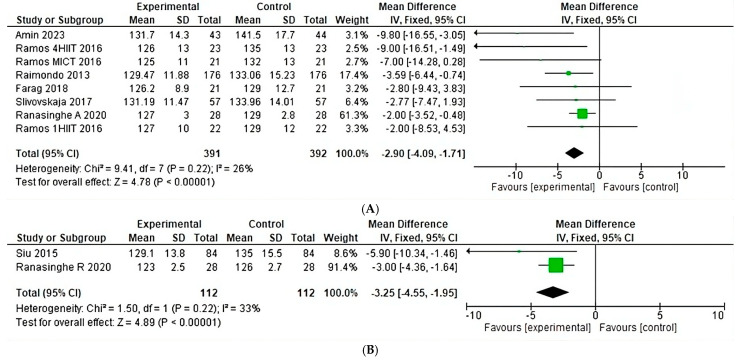
Systolic blood pressure under the effect of different exercises program. (**A**) Systolic blood pressure under the effect of aerobic training. (**B**) Systolic blood pressure under the effect of anaerobic training [22,23,24,25,26,28,30].

With respect to the effect of diastolic blood pressure under different types of exercise, a heterogeneity of 26% was observed (Chi^2^ = 9.41, *p* = 0.22) (Figure 9A) in eight aerobic studies, where the greatest weight is found in the study of Ranasinghe; on the other hand, in two anaerobic studies, a heterogeneity of 33% was observed (Chi^2^ = 1.50, *p* = 0.22) (Figure 9B).

**Waist circumference**

**Figure 10:**
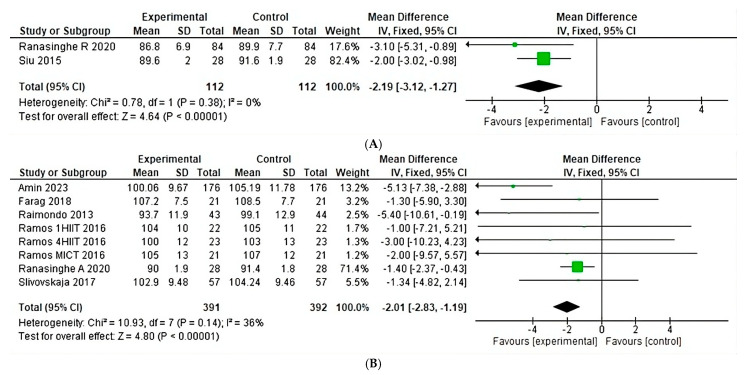
Waist circumference under the effect of different exercises program. (**A**) Waist circumference under the effect of anaerobic training. (**B**) Waist circumference under the effect of aerobic training [22,23,24,25,26,28,30].

After analyzing the effect of anaerobic training conditions on waist circumference, a heterogeneity of 0% is shown (Chi^2^ = 0.78, *p* = 0.38) (Figure 10A) in two studies, with a greater weight found in studies by Siu, and in eight studies on aerobic training conditions a heterogeneity of 36% is shown (Chi^2^ = 10.93, *p* = 0.14) (Figure 10B), with a greater weight found in studies by Ranasinghe. There are not, however, enough anaerobic studies to perform an anaerobic analysis.

**Triglycerides**

**Figure 11:**
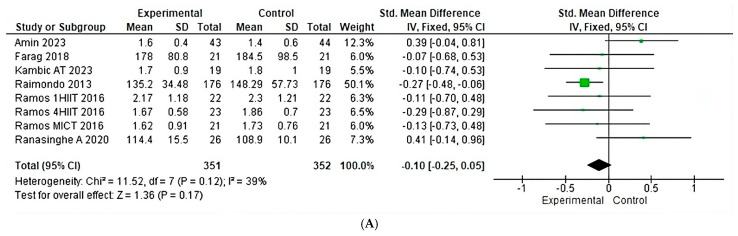

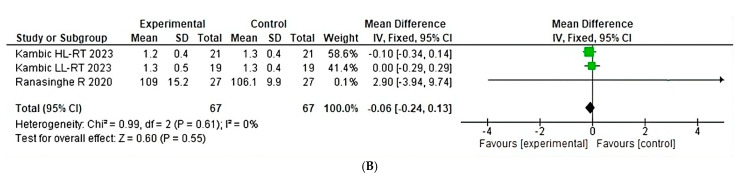
Triglycerides under the effect of different exercises program. (**A**) Triglycerides under the effect of aerobic training. (**B**) Triglycerides under the effect of anaerobic training [24,25,26,27,28,30].

Analyzing the effects of aerobic training conditions on triglycerides shows a heterogeneity of 39% (Chi^2^ = 11.52, *p* = 0.12)(Figure 11A) in eight studies, with a greater weight found in studies by Raimondo. For eight studies of anaerobic training conditions a heterogeneity of 0% is shown (Chi^2^ = 0.99, *p* = 0.61)(Figure 10B); however, there not enough concurrent studies to perform an analysis.

## 4. Discussion

The main objective of this systematic review with meta-analysis was to analyze the effectiveness of the changes of the biochemical, physiological, and anthropometric indicators of MetS under the characteristics of physical exercise and to determine the efficacy associated with the control of indicators that have a greater incidence in the population. The main results of the review are that regardless of the type of exercise, a reduction in the indicators of metabolic syndrome is possible; however, from the analysis carried out, in the indicators of systolic blood pressure, triglycerides and waist circumference, after evaluating a total of 1427 patients, sufficient statistical power was not observed to show such effects.

In a meta-analysis carried out by Wewege et al. (2018) [13] a comparison between types of exercise was carried out, including aerobic exercise, anaerobic exercise, and a combination of both, using MetS indicators as a reference, with the peculiarity of excluding type 2 diabetes mellitus from the review. Thus, when observing the results, aerobic exercise stood out with better benefits, which had a greater effect on women and people over 50 years of age, considering that sex and age can be a modification in response to exercise, where the duration of the programs is equal to or greater than 12 weeks and the frequency is three times a week.

Likewise, another review with meta-analysis carried out in 2021 by Liang et al. [14] whose objective and indicators are similar to the previous meta-analysis, obtained different results, reflecting that anaerobic exercise led the table of benefits compared to aerobic exercise, where the leading benefited indicators were those related to body composition, according to the structures of the applied programs, which followed the guidelines of recommendations established by international organizations such as the ACSM. While both meta-analyses yielded beneficial results, they emphasize that combining moderate-intensity, low-volume exercise for endurance training and long-duration aerobic training has greater impact and significance so that the most precise response may lie in the dose response of concurrent exercise.

When analyzing the results of MetS indicators in the reviewed meta-analyses and the present one, similarities were found concerning the decrease in lipid profile, body composition, and change in diastolic pressure. In the three meta-analyses, 10 different indicators were analyzed, showing significant favorable effects on four to seven indicators, with blood glucose being the indicator with the highest modal value.

On the other hand, Wewege et al., 2018 [13], and Liang et al., 2021 [14], agree on the significance of triglyceride and waist circumference indicators, while Wewege et al., 2018 [13], and this meta-analysis agree on the high-density lipoprotein and diastolic blood pressure indicators. It is worth noting that the present meta-analysis found significance in the total body weight and total cholesterol indicators.

When analyzing the data collected in the present meta-analysis, articles A1, A5, A6, A12, A14, A15, and A18 contrast with most studies. Firstly, all are aerobic-type exercise with times greater than 30 min so that within the metabolic indicators, similarities were found in the favorable effects in relation to total cholesterol, high- and low-density lipoproteins, and fasting blood glucose. One possible answer may be that the post-exercise serum metabolome is different according to exercise modalities, highlighting prominent amino acid, nucleotide, and carbohydrate signaling after endurance exercise compared to lipid-derived enrichment. Circulating availability of no-esterified fatty acids (NEFAs) increases during longer sessions of low- to moderate-intensity physical exercise due to increased release from adipose tissue upon lipoprotein affinity for hydrolysis by lipoprotein lipases (LPLs). NEFA utilization is enhanced by the contraction of fatty acid transporters, which have variable capacities to increase NEFA uptake and oxidation in muscle so that at intensities that cause peak fat oxidation (60–65% of VO2max), the contribution of NEFAs and intracellular lipids is 1:1, approaching their total carbohydrate utilization, so these signaling events increase muscle lipolysis rates, and type I and II fiber lipids can be reduced by 45% and 20%, respectively. In relation to fasting glycemia, this may be because in order for glucose to be oxidized in muscle, the lactate produced simultaneously must first be converted to pyruvate. Glucose oxidation is greater and increasing depending on exercise intensity, dietary carbohydrate, and muscle glycogen levels [32].

Articles A6 and A12 do not show a specification of the intensity of aerobic work during the intervention, so the specificity of the exercise dose may be imprecise; a similar situation exists with A1, where they used a home exercise program through an application, which does not have clarity in the work intensities, so the difference in means is affected, unlike A14 and A15, which describe both aerobic and anaerobic training, long-duration sessions of moderate intensities but infrequent, determined by previous studies, in which both in phase 1 and phase 3 of aerobic training, as in the rest of the studies in the meta-analysis, better metabolic benefits can be obtained.

## 5. Conclusions

The results of this study show that routine exercise prescription is an effective strategy for modulating various biochemical, physiological, and anthropometric indicators related to MetS in adults. From an evidence-based perspective within exercise science, it is confirmed that aerobic, anaerobic, and concurrent training generate distinct physio-logical adaptations, the magnitude and specificity of which depend on the design and manipulation of training variables within the conceptual framework of the FITT-Volume and Progression principle.

Aerobic training was notable for inducing significant improvements in total cholesterol, which reinforces its priority inclusion in intervention programs for the control of MetS. Concurrent protocols, on the other hand, showed greater effectiveness in optimizing lipid profiles, especially HDL levels, fasting blood glucose, diastolic blood pressure, body mass index, and total body weight, suggesting a better efficacy on METs indicators.

Limitations of the study: Some of this study’s limitations include the heterogeneity among the included studies, despite having been critical of the inclusion criteria, as well as the population characteristics and duration of the intervention. On the other hand, the methodological bias of only the lead author conducting the initial search is a major concern.

## Figures and Tables

**Table 1:** Results of methodological quality. Green = available information; Red = information not available.

Cite	Item 1	Item 2	Item 4	Item 8	Item 9	Item 10	Item 11	Total
Raimondo et al. (2013) [26]	X			X	X	X	X	5
Sui el al. (2015) [23]	X	X	X	X	X	X	X	7
Ramos et al. (2016) [25]	X	X	X		X	X	X	6
Slivovskaja et al. (2017) [22]	X				X	X	X	4
Farag et al. (2018) [28]	X	X	X		X	X	X	6
Ranasinghe et al. (2020) [24]	X	X	X	X	X	X	X	7
Bönhof et al. (2022) [29]	X		X	X	X	X		6
Moravcová et al. (2022) [21]	X	X	X		X	X	X	6
Amin et al. (2023) [30]	X	X	X		X	X	X	6
Kambic et al. (2023) [27]	X	X	X		X	X	X	6

**Table 2:** Characteristics of the included studies.

Cite and Location	ID	Objective	Population Characteristics	Exercise Characteristics	Results
Amin et al., 2023, Ghana [30]	A1	To determine the effect of a 12-week culturally appropriate home-based physical activity program on metabolic syndrome markers and quality of life in Ghanaian adults with T2DM.	n = 44 Age: 56.5 ± 8.5 years Sex: both genders Pathology: MetS	Program: resistance band Frequency: 3 sessions/week Intensity: brisk pace, 5.2–5.6 km/h Type: aerobic Time: 45 min	=TG -FBG * =HDL -SBP * -DBP -WC *
Moravcová et al., 2022, Czech Republic [21]	A2	This is to evaluate whether the effect of using Vitadio is comparable to that of participating in an intensive individualized weight reduction program administered in person at a specialized clinic.	n = 29 Age: 43 ± 9.5 years Sex: both genders Pathology: obesity, T2DM	Program: Vitadio app Frequency: daily Intensity: 3 months intensive, 3 months sustaining Type: not specified Time: 60 min	-BW * -BMI * -WC * -FBS * -TG * +HDL * =LDL
Ramos et al., 2016, Australia [25]	A3	To investigate the impact of moderate-intensity continuous training and different volumes of HIIT on circulating intact proinsulin concentration.	n = 23 Age: 57 ± 9 years Sex: both genders Pathology: MetS	Program: 1HIIT (16 weeks) Frequency: 3 times/week Intensity: 85–95% HRpeak Type: aerobic Time: 17 min/session	-BW -BMI -WC * =FBS +HDL * -TG -SBP * -DBP
Ramos et al., 2016, Australia [25]	A4	To investigate the impact of moderate-intensity continuous training and different volumes of HIIT on circulating intact proinsulin concentration.	n = 22 Age: 56 ± 10 years Sex: both genders Pathology: MetS	Program: 4HIIT (16 weeks) Frequency: 3 times/week Intensity: 85–95% HRpeak Type: aerobic Time: 38 min/session	-BW -BMI -WC +FBS +HDL -TG -SBP -DBP *
Ramos et al., 2016, Australia [25]	A5	To investigate the impact of moderate-intensity continuous training and different volumes of HIIT on circulating intact proinsulin concentration.	n = 21 Age: 58 ± 7 years Sex: both genders Pathology: MetS	Program: MICT (16 weeks) Frequency: 5 times/week Intensity: 60–70% HRpeak Type: aerobic Time: 30 min/session	-BW =BMI -WC * -FBS +HDL -TG -SBP * -DBP *
Raimondo et al., 2013, Italy [26]	A6	The study was to assess the metabolic and anti-inflammatory effects of a home-based program of fast walking in patients affected by metabolic syndrome.	n = 95 Age: 59.1 ±13.6 years Sex: both genders Pathology: MetS	Program: fast walking Frequency: 5 times/week Intensity: higher than comfortable Type: aerobic Time: 60 min/session	-BW * -WC * -BMI * -FBS * +HDL * -TC * -TG * -SBP * -DBP *
Balducci et al., 2008, Italy [31]	A7	To evaluate the efficacy of an intensive lifestyle intervention on modifiable cardiovascular disease (CVD) risk factors in a large cohort of people with type 2 diabetes and metabolic syndrome	n = 303 Age: 60 ± 6 years Sex: both genders Pathology: MetS, T2DM, CVD	Program: training program (12 months) LI Frequency: 2 times/week Intensity: 55% (HRpeak); 60% (2 sets, 15 rep) Type: aerobic, resistance Time: 75 min	-TG * -LDL * +HDL * -SBP -DBP -BMI -WC *
Balducci et al., 2008, Italy [31]	A8	To evaluate the efficacy of an intensive lifestyle intervention on modifiable cardiovascular disease (CVD) risk factors in a large cohort of people with type 2 diabetes and metabolic syndrome	n = 303 Age: 60 ± 6 years Sex: both genders Pathology: MetS, T2DM, CVD	Program: training program (12 months) HI Frequency: 2 times/week Intensity: 70% (HRpeak); 80% (3 sets, eight rep) Type: aerobic, resistance Time: 75 min	-TG * -LDL * +HDL * -SBP -DBP -BMI -WC *
Kambic et al., 2023, Slovenia [27]	A9	To examine whether the addition of high-load (HL) or low-load (LL) RT has any effect on the levels of insulin resistance and lipids versus aerobic training (AT) alone in patients with coronary artery disease (CAD).	n = 26 Age: 61 ± 8 years Sex: both genders Pathology: coronary artery disease (CAD)	Program: cycling, HL-RT (12 weeks) Frequency: 3 times/week Intensity: 50–80% HRpeak, 3 sets 70% of 1RM (6–11 rep/set) to 80% of 1RM (12–16 rep/set) Type: aerobic, anaerobic Time: 50 min/session	-BW -BMI * -LDL * -TG +FBG -TC * =HDL
Kambic et al., 2023, Slovenia [27]	A10	To examine whether the addition of high-load (HL) or low-load (LL) RT has any effect on the levels of insulin resistance and lipids versus aerobic training (AT) alone in patients with coronary artery disease (CAD).	n = 28 Age: 61 ± 8 years Sex: both genders Pathology: coronary artery disease (CAD)	Program: cycling, LL-RT (12 weeks) Frequency: 3 times/week Intensity: 50–80% HRpeak, 3 sets 35% of 1RM (6–11 rep/set) to 40% of 1RM (12–16 rep/set) Type: aerobic, anaerobic Time: 50 min/session	-BW -BMI * -LDL =TG -FBG -TC =HDL
Bönhof et al., 2022, Germany [29]	A11	To determine which modality of exercise training as a component of lifestyle intervention may exert favourable effects on somatosensory and autonomic nerve tests in people with type 2 diabetes.	n = 23 Age: 30–65 years Sex: men Pathology: T2DM	Program: HIIT (12 weeks) Frequency: 3 times/week Intensity: 4 min 90% HRpeak, 3 min 70% HRpeakType: aerobic Time: 35 min/session	=BMI -SBP * -DBP * -TG +TC +HDL -LDL
Farag et al., 2018, Iran [28]	A12	In the present study, we hypothesized that the intake of vitamin D and/or C with endurance physical activity might reduce the risk of metabolic syndrome.	n = 180 Age: between 30 and 50 years Sex: both genders Pathology: MetS	Program: climbing, running Frequency: 2 times/week each Intensity: not specified Type: aerobic Time: 360 min/week	-BW -BMI +WC -FBS * -TG * -LDL * +HDL * -SBP -DBP -TC *
Siu et al., 2015, China [23]	A13	To examine the effects of 1 year of yoga exercise on the cardiovascular risk factors, including central obesity, hypertension, dyslipidemia and hyperglycemia in middle-aged and older Hong Kong Chinese adults with MetS.	n = 182 Age: 56 ± 9.1 years Sex: not specified Pathology: MetS	Program: yoga (1 year) Frequency: 3 times/week Intensity: not specified Type: aerobic, anaerobic Time: 60 min/session	-WC * -SBP -DBP -FBS -TG =HDL
Ranasinghe et al., 2020, Australia [24]	A14	To examine the effects of aerobic training (AT) and resistance training (RT) compared to standard care on glycemic control in South Asian Sri Lankan adults with type 2 diabetes mellitus (T2DM).	n = 28 Age: 35–65 years Sex: both genders Pathology: T2DM	Program: aerobic training (3 months) Frequency: 2 times/week Intensity: 60–70% HRpeak Type: aerobic Time: 75 min/session	=BW =BMI +WC -HDL -LDL +TG -SBP =DBP -FBS -TC
Ranasinghe et al., 2020, Australia [24]	A15	To examine the effects of aerobic training (AT) and resistance training (RT) compared to standard care on glycemic control in South Asian Sri Lankan adults with type 2 diabetes mellitus (T2DM).	n = 28 Age: 35–65 years Sex: both genders Pathology: T2DM	Program: resistance training (3 months) Frequency: 2 times/week Intensity: 50%-1RM (8 rep/three sets) Type: anaerobic Time: 75 min/session	-BW =BMI +WC +HDL * -LDL * +TG -SBP +DBP +FBS *
Farag et al., 2019, Iran [28]	A16	To determine the effects of vitamin D supplementation along with endurance physical activity on lipid profile among metabolic syndrome patients.	n = 90 Age: 30–50 years Sex: both genders Pathology: MetS	Program: climbing, running Frequency: 2 times/week each Intensity: not specified Type: aerobic Time: 360 min/week	-TG +TC +LDL +HDL
Slivovskaja et al., 2017, Lithuania [22]	A17	To evaluate if 4-week supervised aerobic training had any impact on anthropometric, metabolic, hemodynamic, and arterial wall parameters in MetS subjects.	n = 57 Age: 52.79 ± 6.65 years Sex: both genders Pathology: MetS	Program: (4 weeks) Frequency: 5 times/week Intensity: gradually increased by 40–60% HRRType: aerobic training Time: 30–40 min/session	-BM -WC * -LDL * -DBP * -BP *
Kambic et al., 2023, Slovenia [27]	A18	Examine whether the addition of high-load (HL) or low-load (LL) RT has any effect on insulin resistance and lipid levels compared to aerobic training (AT) alone in patients with coronary artery disease (CAD).	n = 25 Age: 61 ± 8 years Sex: both genders Pathology: Coronary artery disease (CAD).	Program: Cycling, HL-RT (12 weeks) Frequency: 3 times/week Intensity: 50–80% HRpeak, Type: aerobic Time: 40 min/session	-BW -LDL =TG =FBG -TC =HDL

MetS = metabolic syndrome; T2DM = type 2 diabetes mellitus; CAD = coronary artery disease; CVD = cardiovascular disease; BW = body weight; BM = body mass; BMI = body mass index; WC = waist circumference; SBP = systolic blood pressure; DBP = diastolic blood pressure; BP = blood pressure; TG = triglycerides; FBS = fasting blood sugar; HDL = high-density lipoprotein; LDL = low-density lipoprotein; VO2max = maximum rate of oxygen consumption attainable during physical exertion. +Increases, -reduces, = no change, * significant.

## Data Availability

The raw data supporting the conclusions of this article will be made available by the authors on request.

## Abbreviations

The following abbreviations are used in this manuscript:

MetS	Metabolic syndrome
T2DM	Type 2 diabetes mellitus
HDL	High-density lipoprotein
LDL	Low-density lipoprotein
CVD	Cardiovascular disease
GDP	Gross domestic product
PEDro	Physiotherapy evidence database
WMD	Weighted mean difference
CI	Confidence interval
FITT	Frequency, intensity, type, time
HIIT	High-intensity interval training
HRpeak	High-rate peak
CAD	Coronary artery disease
BW	Body weight
BM	Body mass
BMI	Body mass index
WC	Waist circumference
SBP	Systolic blood pressure
DBP	Diastolic blood pressure
BP	Blood pressure
TG	Triglycerides
FBS	Fasting blood sugar
